# Supplementary material for: Trends in Excess Winter Mortality (EWM) from 1900/01 to 2019/20—Evidence for a Complex System of Multiple Long-Term Trends
Source: Int J Environ Res Public Health. 2022 Mar 14;19(6):3407. doi: 10.3390/ijerph19063407 (PMC8953800; doi:10.3390/ijerph19063407)
Supplement: Supplementary file 1 [file ijerph-19-03407-s001.zip › ijerph-1602749-supplementary.pdf]

Supplementary Material

Table S1. Summary of data relating to each country included in the influenza vaccine doses distributed analysis. The column "10% achieved" gives the winter at which 10% population vaccination coverage was achieved.

Country	Latitude	EWM adjustment	Data (n) up to 2013/14	Vaccination	
				Range	10% achieved
**Singapore**	**1.3**	**1.650**	**34**	**0%-10%**	2009/10
Hong Kong	22.3	0.656	33	1%-15%	2003/04
South Korea	36.1	1.463	23	5%-41%	1996/97
Japan	36.3	0.668	33	0%-51%	2000/01
USA(!)	38.0	1.000	40	6%-53%	1988/89
Turkey	39.1	0.981	4	3%-4%	No
Portugal	39.6	0.443	34	1%-25%	1994/95
Greece	39.7	1.150	14	12%-32%	2000/01
Armenia	40.3	0.471	22	0%	No
Spain	40.4	0.590	34	5%-34%	1988/89
Macedonia	41.4	0.826	21	0%-1%	No
Kyrgyzstan	41.5	1.600	8	0%	No
Uzbekistan	41.8	0.979	7	0%-1%	No
Italy	42.5	0.746	24	15%-34%	1984/85
Montenegro	42.8	0.993	9	0%-7%	No
Bosnia and Herzegovina	43.7	0.939	10	0%-4%	No
San Marino	43.9	0.453	26	0%-15%	2004/05
France	46.6	1.080	34	9%-36%	1988/89
Switzerland	46.8	0.900	34	5%-22%	1998/99
Austria	47.6	0.996	34	1%-17%	1999/00
Kazakhstan	48.1	1.520	21	2%-4%	No
Ukraine	48.4	1.060	30	0%-2%	No
Luxembourg	49.6	0.924	12	15%-48%	2004/05
Belgium	50.6	0.854	34	5%-27%	1993/94
Germany	51.9	1.200	34	6%-35%	1997/98
Poland	52.1	1.040	34	1%-10%	2001/02
Netherlands(α)	52.3	1.070	44	3%-46%	1994/95
Ireland	53.2	0.738	22	3%-31%	2002/03
UK	54.6	0.760	26	2%-33%	1995/96
Canada	56.1	1.090	34	4%-38%	1990/91
Denmark	56.1	1.100	32	0%-26%	1998/99
Latvia	56.9	1.095	25	1%-14%	2005/06
Estonia	59.4	1.120	34	1%-7%	No
Sweden	62.7	0.990	34	0%-21%	2001/02
Finland(α)	64.3	1.240	44	2%-34%	1995/96
Iceland	64.9	0.935	34	1%-28%	1986/87
Norway	66.8	1.020	34	1%-16%	1995/96

(!) Data for USA extended to 2019/20 due to doses data available from US CDC. (α) Low vaccination at the beginning of the time series enables extrapolation back in time using an assumed linear trend.

Table S2. Summary of data relating to each country (n=98) included in the proportion elderly vaccinated study.

Country	EWM Adjustment	Median EWM	Data (n=)	Age 65+ vaccinated (%)		
				Minimum	Maximum	Median
Albania	0.44	31.2%	13	0%	14%	6%
Armenia	0.47	24.6%	15	0%	2%	0%
Australia	0.72	16.6%	32	12%	79%	58%
Austria	0.87	13.8%	33	0%	49%	21%
Azerbaijan	0.79	14.9%	21	0%	0%	0%
Bahamas	0.99	12.1%	22	0%	27%	0%
Barbados	0.87	13.8%	6	0%	40%	8%
Belarus	1.38	9.4%	18	5%	76%	23%
Belgium	0.72	15.4%	33	15%	65%	57%
Bermuda	0.62	19.6%	17	0%	12%	0%
Bosnia and Herzegovina	0.97	12.4%	10	0%	0%	0%
Bulgaria	0.71	17.6%	20	2%	20%	8%
Canada	1.03	11.6%	33	25%	71%	60%
Chile	0.57	21.2%	32	3%	68%	18%
Costa Rica	1.54	8.2%	26	0%	50%	15%
Croatia	0.78	16.0%	31	0%	43%	23%
Cuba	1.22	9.7%	26	0%	99%	28%
Cyprus	0.52	22.0%	18	23%	49%	33%
Czechia	1.08	11.4%	28	0%	24%	17%
Denmark	1.08	11.1%	33	4%	52%	20%
Egypt	0.90	13.4%	25	0%	1%	0%
El Salvador	1.20	10.0%	23	0%	42%	12%
England	0.68	17.6%	33	12%	75%	71%
Estonia	1.10	10.9%	32	0%	20%	2%
Finland	1.12	10.7%	33	4%	52%	40%
France	0.87	13.7%	33	39%	67%	52%
Georgia	0.63	18.9%	16	0%	0%	0%
Germany	1.00	12.0%	33	13%	63%	38%
Greece	1.04	11.0%	20	20%	59%	49%
Greenland	0.66	18.1%	28	0%	0%	0%
Guadeloupe	1.18	10.1%	6	0%	18%	9%
Guam	0.79	15.3%	11	0%	0%	0%
Guatemala	2.42	5.0%	9	1%	15%	9%
Hong Kong SAR	0.67	20.2%	33	0%	44%	5%
Hungary	1.03	11.7%	32	13%	39%	27%
Iceland	1.01	11.5%	31	35%	49%	43%
Ireland	0.75	15.8%	20	38%	70%	59%

Country	EWM Adjustment	Median EWM	Data (n=)	Age 65+ vaccinated (%)		
				Minimum	Maximum	Median
Israel	0.57	21.4%	27	43%	66%	49%
Italy	0.75	16.0%	29	40%	68%	52%
Japan	0.64	18.9%	33	0%	57%	48%
Kazakhstan	1.54	7.6%	18	1%	15%	3%
Kosovo	0.80	15.0%	9	0%	0%	0%
Kuwait	0.75	17.0%	28	0%	23%	4%
Kyrgyzstan	1.76	6.8%	13	0%	7%	1%
Latvia	1.01	12.1%	31	0%	15%	3%
Lebanon	0.63	18.9%	20	5%	35%	15%
Liechtenstein	0.47	25.6%	32	20%	30%	27%
Lithuania	0.95	12.7%	26	0%	25%	9%
Luxembourg	0.93	13.0%	30	15%	54%	40%
Macao SAR	0.57	21.0%	31	9%	36%	25%
Malaysia	2.13	5.6%	24	0%	1%	0%
Maldives	0.89	13.6%	24	0%	6%	0%
Malta	0.37	34.0%	30	20%	57%	46%
Martinique	1.43	9.0%	11	0%	6%	0%
Mauritius	0.86	13.9%	26	0%	51%	14%
Mexico	0.74	15.9%	27	1%	88%	51%
Mongolia	1.16	10.4%	15	0%	15%	0%
Montenegro	0.89	13.5%	14	14%	31%	17%
Netherlands	0.89	13.4%	32	25%	84%	70%
New Caledonia	0.83	14.6%	23	7%	35%	21%
New Zealand	0.67	18.4%	33	9%	69%	58%
North Macedonia	0.83	14.7%	22	0%	12%	4%
Northern Ireland	0.71	17.0%	31	12%	78%	72%
Norway	0.96	12.4%	33	15%	44%	31%
Philippines	1.77	6.8%	16	0%	9%	2%
Poland	1.09	11.7%	33	0%	32%	8%
Portugal	0.44	27.4%	33	0%	65%	42%
Puerto Rico	1.18	10.2%	22	0%	37%	8%
Qatar	0.88	13.2%	23	0%	59%	5%
Republic of Moldova	0.66	18.3%	20	0%	5%	3%
Reunion	1.03	12.5%	10	0%	0%	0%
Romania	0.71	17.3%	32	0%	29%	15%
Russian Federation	1.79	6.7%	23	1%	69%	14%
Saint Lucia	0.78	15.3%	4	0%	0%	0%
Saint Vincent & Grenadines	0.73	16.1%	17	0%	20%	0%
Scotland	0.81	14.8%	30	12%	77%	72%
Serbia	0.95	12.7%	19	0%	15%	9%

Country	EWM Adjustment	Median EWM	Data (n=)	Age 65+ vaccinated (%)		
				Minimum	Maximum	Median
Singapore	1.56	7.4%	31	2%	21%	8%
Slovakia	1.32	9.1%	29	0%	38%	14%
Slovenia	0.83	14.2%	32	0%	35%	13%
South Africa	0.95	12.6%	18	0%	17%	3%
South Korea	1.46	8.2%	27	9%	86%	73%
Spain	0.60	20.0%	33	37%	70%	58%
Sri Lanka	1.12	10.7%	13	0%	0%	0%
Suriname	1.31	9.1%	16	0%	0%	0%
Sweden	0.94	12.7%	33	13%	66%	44%
Switzerland	0.82	14.6%	32	14%	61%	36%
Trinidad and Tobago	1.44	9.6%	17	0%	25%	2%
Turkey	0.82	14.6%	21	1%	13%	6%
Ukraine	1.10	11.5%	27	0%	70%	0%
United Kingdom	1.00	12.0%	33	12%	75%	71%
Uruguay	0.42	30.5%	18	0%	70%	0%
US Virgin Islands	0.86	15.0%	7	0%	16%	2%
USA	1.00	12.0%	33	36%	71%	65%
Uzbekistan	1.30	9.2%	12	0%	13%	0%
Venezuela	1.59	8.0%	13	0%	50%	15%
Wales	0.74	16.1%	20	9%	69%	60%

Table S3. Example of high/Low behaviour in EWM for american states over the 13 winter seasons ending 2019/20. Winter of 2019/20 was truncated at March to avoid the effects of COVID-19. High/low groups are by visual inspection due to the gap between the range for high and low values.

State	High/Low	EWM Range	Frequency
USA	High	14%-16%	6
	Low	7%-10%	7
Arizona	High	11%-16%	8
	Low	6%-10%	5
Alaska	High	9%-15%	7
	Low	5%-6%	6
New Jersey	High	14%-16%	7
	Low	8%-10%	6
New York	High	14%-16%	6
	Low	6%-10%	7
Ohio	High	14%-18%	5
	Low	8%-10%	8
Texas	High	14%-16%	5
	Low	9%-11%	8
Wyoming	High	9%-14%	7
	Low	4%-8%	6
Wisconsin	High	10%-16%	8
	Low	6%-9%	5
West Virginia	High	10%-16%	10
	Low	6%-8%	3

Figure S1. Relationship between median EWM among American states and proportion of persons aged 65+ who are obese

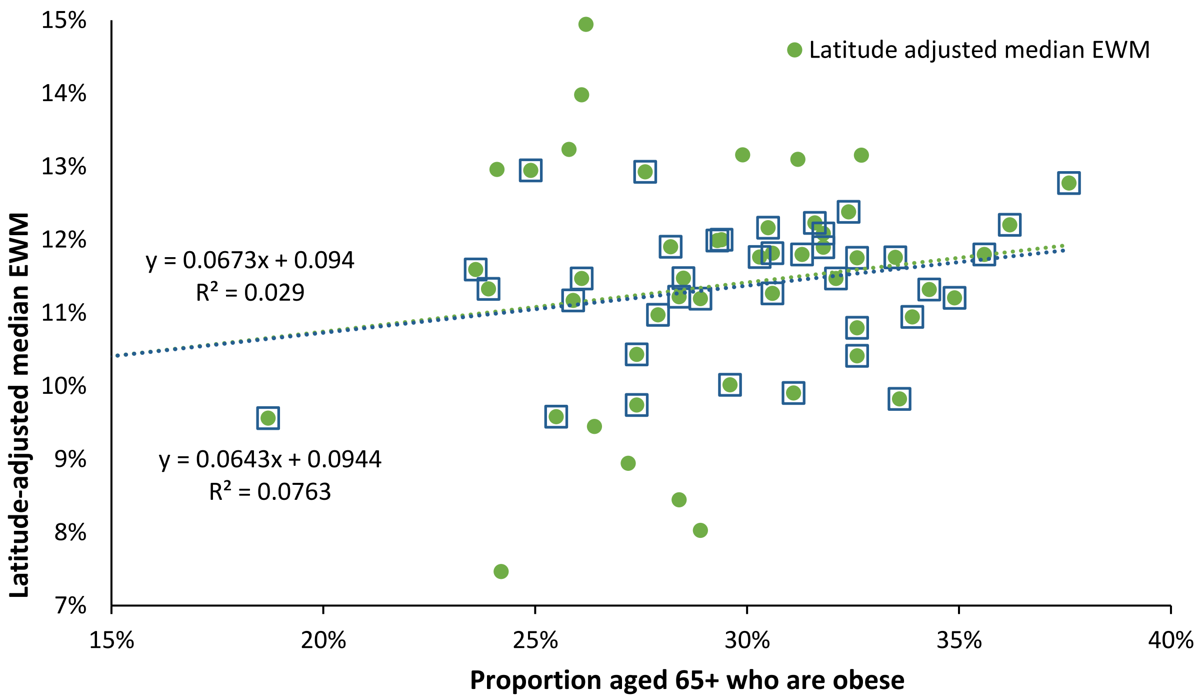

Footnote: Data regarding obesity in those aged 65+ in 2020 was from DNPAO Data, Trends and Maps: Explore by Topic | CDC. The slope has been calculated using all states or after trimming high/low EWM states. As can be seen trimming has negligible effect on the observed slope.

Figure S2. Trend in EWM for Denmark, Iceland, Singapore, and Sweden over the 60-year period 1960 to 2020

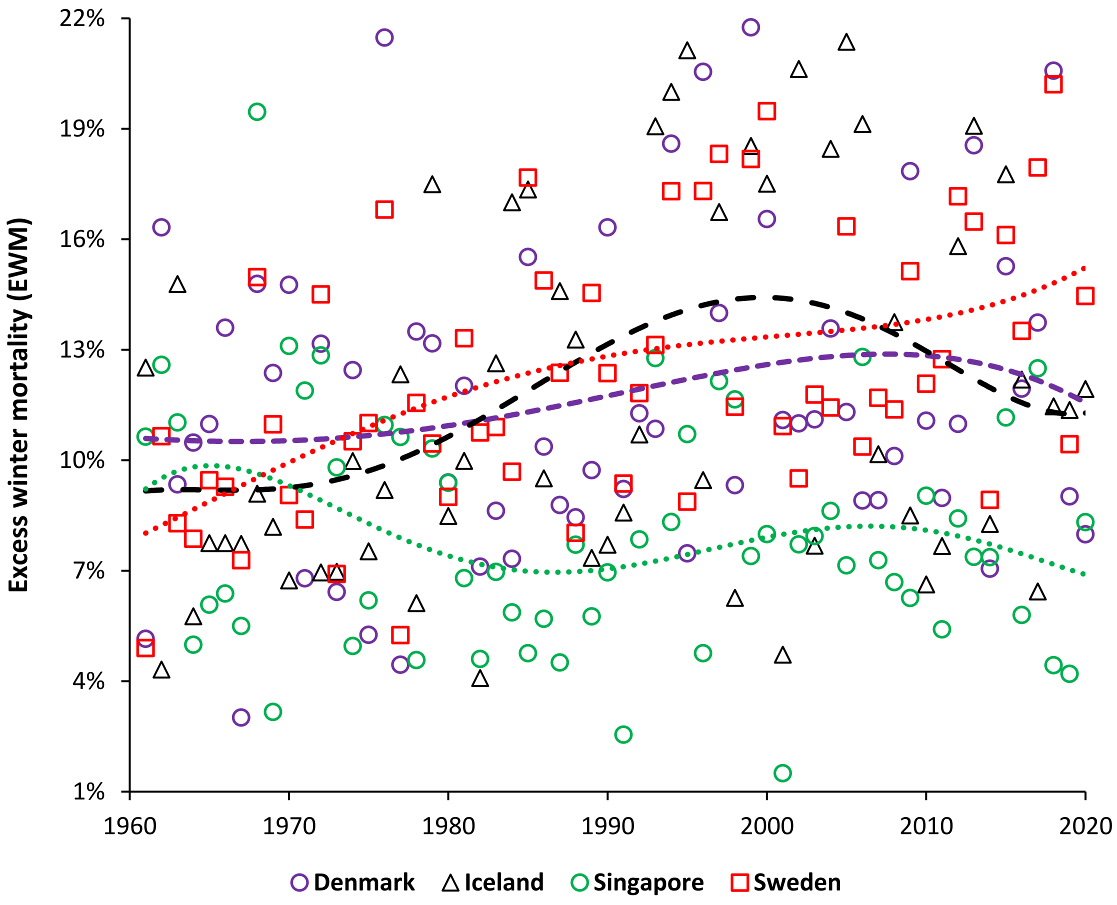

Footnote: The trend for each country is estimated using a 4th or 5th order polynomial. Singapore (closest to the equator) seems to have a slight downward trend while Sweden appears to have the highest trend upward. During the period 1992 to 2010 obesity among Singaporeans aged 60 – 69 was static [60]. Singapore has traditionally been a low influenza vaccination country. The trends are dominated by very high year-to-year volatility which is reflected in the high volatility in Figure 6. Since this study ran from 1980 onward it is considered that adjustment for the underlying trends relative to the USA would make only a small impact on the observed volatility in Figure 6. The non-linear trends are consistent with the overall observation of this paper that long-term trends in EWM are regulated by complex factors.

Figure S3. US CDC estimated flu deaths (adjusted for growth in deaths over time) versus EWM.

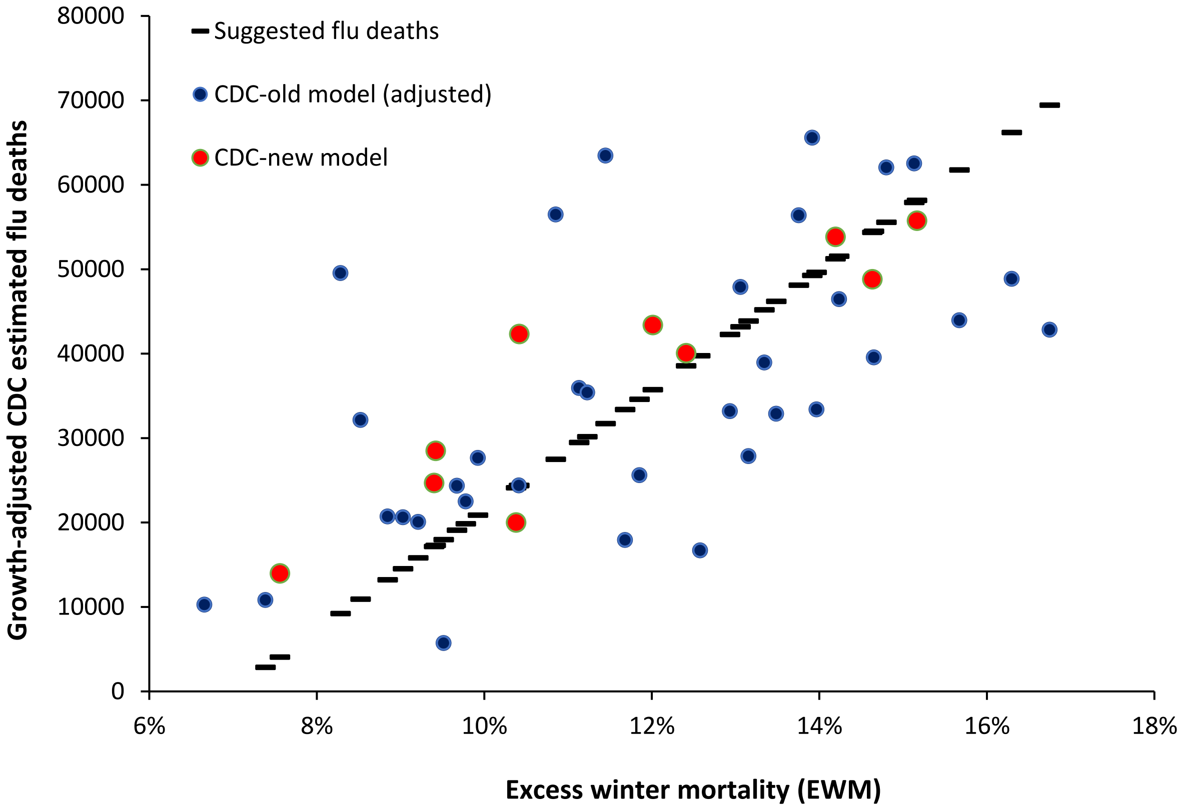

Footnote: CDC estimated flu deaths were first adjusted for the growth in total deaths over time. Data is from [75-77]. The underlying trend in total deaths was determined from a series of polynomial curve fits giving an adjustment factor for each year taking flu deaths to the 2020 equivalent. Over time the CDC has used two models to estimate flu deaths (old model covers 1976/77 to 2006/07, while the new model is from 2007/08 onward) of which the most recent model gives higher estimated flu deaths [75-77]. Data from the earlier model was adjusted up to the more recent model by plotting both sets of data against EWM and using the difference between the regression lines as the adjustment factor. The line for suggested flu deaths is in line with Figures 5 and A3 and assumes that zero flu deaths occur at an EWM around 7% to 8%. Note the far higher scatter in the old CDC results. The R-squared for the old model is 0.448 while that for the new model is 0.8403. This scatter arises because the CDC uses a limited sample of hospitals from which admissions and other data are used to estimate flu deaths. As a result, there are multiple sampling issues, hidden assumptions, and adjustment factor issues. While the new CDC model has a higher R-squared, the CDC models are less reliable than may be expected and lead to over-estimated influenza deaths.

On average the new CDC model possibly overestimates flu deaths by around 14%. For the old model the high years gives flu deaths which are incompatible with excess winter deaths (EWD).

After adjusting all years for the growth in total deaths in the USA over the period 1957/58 to 2019/20 there was a median of 1.047 million winter deaths (interquartile range 1.027 – 1.063 million), a median excess winter deaths of 108,500 (IQR 89,800 – 130,700), and a median estimated winter influenza deaths of 30,000 (IQR 20,000 – 43,000). Estimated influenza deaths as a proportion of excess winter deaths had a median of 28% (IQR 20% - 36%). The proportion influenza deaths reached a maximum

of 50% of excess winter deaths in the winter of 2001/02, which was not a pandemic year. Hence within these ranges there is ample opportunity for the estimated influenza deaths to “steal” from otherwise unrelated reported cause of death in persons who would otherwise have died in that year.

For the USA, EWM reached a minimum in 2011/12 of 7.6% and the US CDC noted that this winter set a record for the lowest and shortest peak in ILI [78]. Outpatient visits, inpatient admissions and deaths were at baseline levels, i.e., 2011/12 represents the lowest possible influenza year. Other than 3 high years between 1997/98 to 1999/00 (14.2%, 15.7%, 16.8% EWM), EWM reached a maximum of 15.2% during the winter of 2014/15 and 14.2% in 2017/18. Both of the later years saw high influenza outpatient visits, hospitalizations, and deaths [79,80]. Hence EWM is correctly detecting the severity of each influenza season. The CDC estimated 12,000 influenza deaths in 2011/12 and 52,000 in 2017/18 [75-77].

Figure S4. Rolling/moving EWM calculation for Sweden, 1851 to 1920

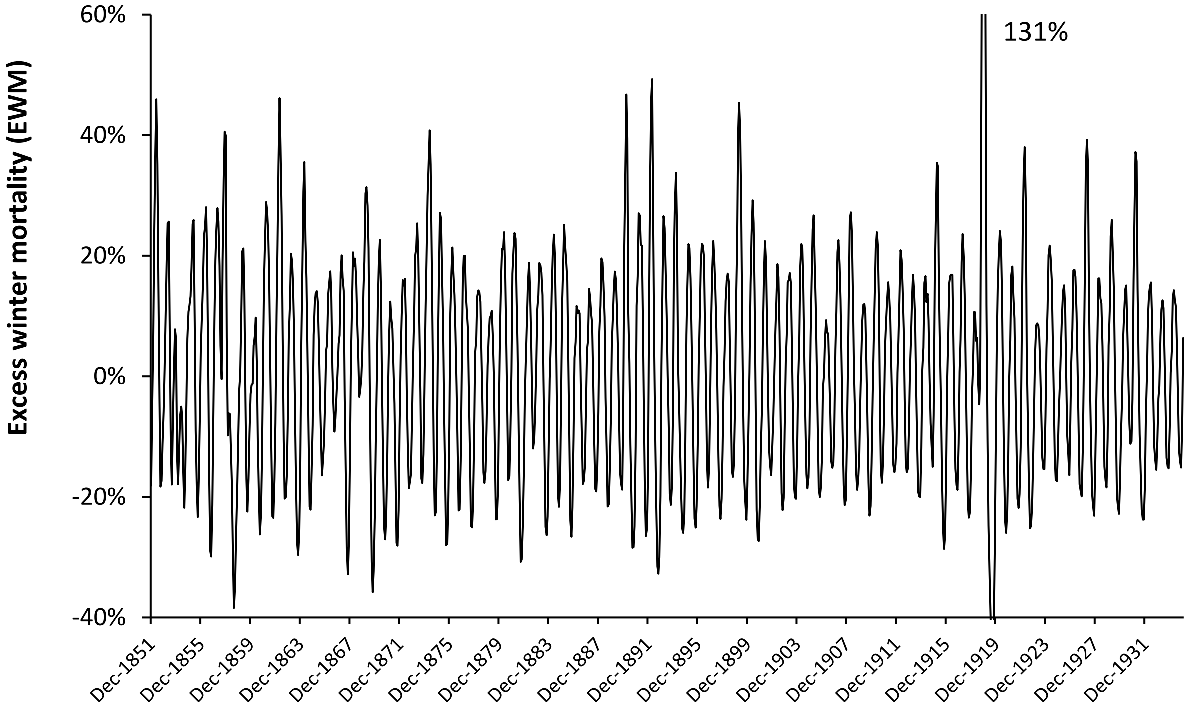

References

1. Jones, R. Excess Winter Mortality (EWM) as a Dynamic Forensic Tool: Where, When, Which Conditions, Gender, Ethnicity and Age. *Int. J. Environ. Res. Public Health* **2021**, *18*, 2161. https://doi.org/10.3390/ijerph18042161.
2. Glezen, W.P. Emerging Infections: Pandemic Influenza. *Epidemiol. Rev.* **1996**, *18*, 64–76. https://doi.org/10.1093/oxfordjournals.epirev.a017917.
3. Meltzer, M.I.; Cox, N.J.; Fukuda, K. The Economic Impact of Pandemic Influenza in the United States: Priorities for Intervention. *Emerg. Infect. Dis.* **1999**, *5*, 659–671. https://doi.org/10.3201/eid0505.990507.
4. Nicoll, A.; Ciancio, B.C.; Chavarrias, V.L.; Mølbak, K.; Pebody, R.; Pedzinski, B.; Penttinen, P.; van der Sande, M.; Snacken, R.; Van Kerkhove, M.D. Influenza-related deaths-available methods for estimating numbers and detecting patterns for seasonal and pandemic influenza in Europe. *Eurosurveillance* **2012**, *17*, 20162. https://doi.org/10.2807/ese.17.18.20162-en.
5. Muscatello, D.J.; Newall, A.T.; Dwyer, D.E.; MacIntyre, C.R. Mortality Attributable to Seasonal and Pandemic Influenza, Australia, 2003 to 2009, Using a Novel Time Series Smoothing Approach. *PLoS ONE* **2013**, *8*, e64734. https://doi.org/10.1371/journal.pone.0064734.
6. EuroMOMO. FluMOMO. FluMOMO—EUROMOMO. Available online: https://www.euromomo.eu/how-it-works/flumomo (accessed on 14 January 2022).
7. Rolfes, M.A.; Foppa, I.M.; Garg, S.; Flannery, B.; Brammer, L.; Singleton, J.A.; Burns, E.; Jernigan, D.; Olsen, S.J.; Bresee, J.; et al. Annual estimates of the burden of seasonal influenza in the United States: A tool for strengthening influenza surveillance and preparedness. *Influenza Other Respir. Viruses* **2018**, *12*, 132–137. https://doi.org/10.1111/irv.12486.
8. Paget, J.; Spreeuwenberg, P.; Charu, V.; Taylor, R.J.; Iuliano, A.D.; Bresee, J.; Simonsen, L.; Viboud, C. Global Seasonal Influenza-Associated Mortality Collaborator Network and GLaMOR Collaborating Teams. Global mortality associated with seasonal influenza epidemics: New burden estimates and predictors from the GLaMOR Project. *J. Glob. Health* **2019**, *9*, 020421. https://doi.org/10.7189/jogh.09.020421.
9. Eurostat, Deaths (Total) by Month. Deaths (Total) by month—Products Datasets—Eurostat (europa.eu). Available online: https://ec.europa.eu/eurostat/en/web/products-datasets/-/DEMO_MMONTH (accessed on 11 November 2021).
10. UNData. Deaths by Month of Death. UNdata | Record View | Deaths by Month of Death. Available online: https://data.un.org/Data.aspx?d=POP&f=tableCode%3a65%3bcountryCode%3a804&c=2,3,6,8,10,12,13,14&s=_countryEnglishNameOrderBy:asc,refYear:desc,areaCode:asc&v=1 (accessed on 11 November 2021).
11. Doshi, P. Trends in Recorded Influenza Mortality: United States, 1900–2004. *Am. J. Public Health* **2008**, *98*, 939–945. https://doi.org/10.2105/AJPH.2007.119933.
12. CDC National Vital Statistics System. Monthly and 12 Month–Ending Number of Live Births, Deaths and Infant Deaths, United States. State and National Provisional Counts (cdc.gov). Available online: https://www.cdc.gov/nchs/nvss/vsrr/provisional-tables.htm (accessed on 11 November 2021).
13. CDC National Vital Statistics System. Deaths by State. State and National Provisional Counts (cdc.gov) Available online: https://www.cdc.gov/nchs/nvss/vsrr/provisional-tables.htm (accessed on 19 February 2022).
14. Statistics Denmark. Live Births and Deaths by Movement and Time. Available online: https://www.statbank.dk/20014 (accessed on 16 April 2021).
15. Statistics Sweden. Deaths by Year and Month. Births and Deaths Per Month by Sex. Year 1851–2021. PxWeb (scb.se) Available online: https://www.statistikdatabasen.scb.se/pxweb/en/ssd/START__BE__BE0101__BE0101G/ManadFoddDod/ (accessed on 19 February 2022).
16. Statistics Singapore. Deaths by Ethnic Group and Sex, Monthly. (DOS) | SingStat Table Builder—Deaths By Ethnic Group and Sex. Available online: https://tablebuilder.singstat.gov.sg/table/TS/M810121 (accessed on 14 January 2022).
17. Statistics Estonia. Monthly Deaths. Deaths | Statistikaamet. Available online: https://www.stat.ee/en/find-statistics/statistics-theme/population/deaths (accessed on 12 December 2021).
18. Fedson, D.S.; Hannoun, C.; Leese, J.; Sprenger, M.J.; Hampson, A.W.; Bro-Jørgensen, K.; Ahlbom, A.M.; Nøkleby, H.; Valle, M.; Olafsson, O.; et al. Influenza vaccination in 18 developed countries, 1980–1992. *Vaccine* **1995**, *13*, 623–627. https://doi.org/10.1016/0264–410x(94)00041–k.
19. Fedson, D.S.; Hirota, Y.; Shin, H.-K.; Cambillard, P.-E.; Kiely, J.; Ambrosch, F.; Hannoun, C.; Leese, J.; Sprenger, M.J.; Hampson, A.W.; et al. Influenza vaccination in 22 developed countries: An update to 1995. *Vaccine* **1997**, *15*, 1506–1511. https://doi.org/10.1016/s0264-410x(97)00091-1.
20. Ambrosch, F.; Fedson, D.S. Influenza Vaccination in 29 Countries. *PharmacoEconomics* **1999**, *16*, 47–54. https://doi.org/10.2165/00019053-199916001-00007.
21. Vanessen, G.; Palache, A.; Forleo, E.; Fedson, D. Influenza vaccination in 2000: Recommendations and vaccine use in 50 developed and rapidly developing countries. *Vaccine* **2003**, *21*, 1780–1785. https://doi.org/10.1016/s0264-410x(03)00072-0.

22. The Macroepidemiology of Influenza Vaccination (MIV) Study Group. The macroepidemiology of influenza vaccination in 56 countries, 1997–2003. *Vaccine* **2005**, *23*, 5133–5143. https://doi.org/10.1016/j.vaccine.2005.06.010.
23. Palache, A. Seasonal influenza vaccine provision in 157 countries (2004–2009) and the potential influence of national public health policies. *Vaccine* **2011**, *29*, 9459–9466. https://doi.org/10.1016/j.vaccine.2011.10.030.
24. Palache, A.; Oriol-Mathieu, V.; Abelin, A.; Music, T. Seasonal influenza vaccine dose distribution in 157 countries (2004–2011). *Vaccine* **2014**, *32*, 6369–6376. https://doi.org/10.1016/j.vaccine.2014.07.012.
25. Palache, A.; Oriol-Mathieu, V.; Fino, M.; Xydia-Charmanta, M. Seasonal influenza vaccine dose distribution in 195 countries (2004–2013): Little progress in estimated global vaccination coverage. *Vaccine* **2015**, *33*, 5598–5605. https://doi.org/10.1016/j.vaccine.2015.08.082.
26. CDC. Historical Reference of Seasonal Influenza Vaccine Doses Distributed. Historical Reference of Seasonal Influenza Vaccine Doses Distributed|CDC. Available online: https://www.cdc.gov/flu/prevent/vaccine-supply-historical.htm (accessed on 24 December 2021).
27. Macrotrends. US Population Growth Rate 1950–2021. MacroTrends. Available online: https://www.macrotrends.net/countries/USA/us/population-growth-rate (accessed on 11 November 2021).
28. Simonsen, L.; Reichert, T.A.; Viboud, C.; Blackwelder, W.C.; Taylor, R.J.; Miller, M.A. Impact of Influenza Vaccination on Seasonal Mortality in the US Elderly Population. *Arch. Intern. Med.* **2005**, *165*, 265–272. https://doi.org/10.1001/archinte.165.3.265.
29. Centers for Disease Control and Prevention (CDC). Influenza Vaccination Coverage Trends, 1989 to 2008. Available online: https://www.cdc.gov/flu/pdf/professionals/nhis89_08fluvaxtrendtab.pdf (accessed on 8 February 2021).
30. CDC. Influenza Vaccine Coverage, Adults Aged 65 Years and Older, United States. Influenza Vaccination Coverage, Adults 65 Years and Older, United States|FluVaxView|Seasonal Influenza (Flu)|CDC. Available online: https://www.cdc.gov/flu/fluvaxview/dashboard/vaccination-coverage-adults-65-over.htm (accessed on 11 November 2021).
31. Centers for Disease Control and Prevention. Flu Vaccination Coverage, United States, 2018–2019 Influenza Season. FluVaxView|Seasonal Influenza (Flu)|CDC. Available online: https://www.cdc.gov/flu/fluvaxview/coverage-1819estimates.htm (accessed on 30 December 2021).
32. CDC. Flu Vaccination Coverage, United States, 2019–2020 Influenza Season. FluVaxView|Seasonal Influenza (Flu)|CDC. Available online: https://www.cdc.gov/flu/fluvaxview/coverage-1920estimates.htm (accessed on 8 February 2021).
33. OECD Data. Influenza Vaccination Rates (Full Indicator Data). Health Care Use—Influenza Vaccination Rates—OECD Data. Available online: https://data.oecd.org/healthcare/influenza-vaccination-rates.htm (accessed on 14 January 2022).
34. Spruijt, I.T.; de Lange, M.M.; Dijkstra, F.; Donker, G.A.; van der Hoek, W. Long-term correlation between influenza vaccination coverage and incidence of influenza-like illness in 14 European Countries. *PLoS ONE* **2016**, *11*, e0163508.
35. WHO. Influenza Vaccination Data Base. Influenza Vaccination Policy (who.int). Available online: https://immunizationdata.who.int/pages/indicators-by-category/influenza.html?ISO_3_CODE=&YEAR= (accessed on 14 January 2022).
36. World Health Organisation. Influenza Vaccination Coverage, Elderly. European Health Information Gateway (who.int). 2020. Available online: https://gateway.euro.who.int/en/indicators/infl_8-influenza-vaccination-coverage-elderly/ (accessed on 8 February 2021).
37. Public Health England. Green Book of Immunization. Chapter 19 Influenza, 2020. The Green Book of Immunisation—Chapter 19 Influenza (publishing.service.gov.uk). Available online: https://assets.publishing.service.gov.uk/government/uploads/system/uploads/attachment_data/file/931139/Green_book_chapter_19_influenza_V7_OCT_2020.pdf (accessed on 12 December 2021).
38. Public Health Wales. Annual Influenza Surveillance and Influenza Vaccination Uptake Reports: 2003–2020. Public Health Wales Health Protection Division. Available online: https://www.wales.nhs.uk/sites3/page.cfm?orgid=457&pid=55714 (accessed on 12 March 2021).
39. Jorgensen, P.; Mereckiene, J.; Cotter, S.; Johansen, K.; Tsolova, S.; Brown, C. How close are countries of the WHO European Region to achieving the goal of vaccinating 75% of key risk groups against influenza? Results from national surveys on seasonal influenza vaccination programmes, 2008/2009 to 2014/2015. *Vaccine* **2018**, *36*, 442–452. https://doi.org/10.1016/j.vaccine.2017.12.019.
40. ECDC. Seasonal Influenza Vaccination in Europe—Vaccination Recommendations and Coverage Rates for Eight Influenza Seasons (2007–2008 to 2014–2015). (europa.eu). 20 July 2017. Available online: https://www.ecdc.europa.eu/en/publications-data/seasonal-influenza-vaccination-europe-vaccination-recommendations-and-coverage-2007-2015 (accessed on 25 February 2022).
41. Blank, P.; Schwenkglenks, M.; Szucs, T.D. The impact of European vaccination policies on seasonal influenza vaccination coverage rates in the elderly. *Hum. Vaccines Immunother.* **2012**, *8*, 328–335. https://doi.org/10.4161/hv.18629.

42. Blank, P.R.; Schwenkglenks, M.; Szucs, T.D. Influenza vaccination coverage rates in five European countries during season 2006/07 and trends over six consecutive seasons. *BMC Public Health* **2008**, *8*, 272. https://doi.org/10.1186/1471-2458-8-272.
43. Korkmaz, P.; Kilit, T.P.; Onbasi, K.; Ozatag, D.M.; Toka, O. Influenza vaccination prevalence among the elderly and individuals with chronic disease, and factors affecting vaccination uptake. *Central Eur. J. Public Health* **2019**, *27*, 44–49. https://doi.org/10.21101/cejph.a5231.
44. Beard, F.; Hendry, A.; Macartney, K. Influenza vaccination uptake in our most vulnerable groups: How well are we protecting them in 2019? *Commun. Dis. Intell.* **2020**, *2020*, 44. https://doi.org/10.33321/cdi.2020.44.27.
45. Newspoll Omnibus Survey. Summary Report Flu Vaccinations Department of Health Newspoll Ref: 140604 23 June 2014. (health.gov.au). Available online: https://www.health.gov.au/sites/default/files/report-newspoll-flu-vaccinations-survey-jun-2014.pdf (accessed on 12 December 2021).
46. Zürcher, K.; Zwahlen, M.; Berlin, C.; Egger, M.; Fenner, L. Losing ground at the wrong time: Trends in self-reported influenza vaccination uptake in Switzerland, Swiss Health Survey 2007–2017. *BMJ Open* **2021**, *11*, e041354. https://doi.org/10.1136/bmjopen-2020-041354.
47. Singapore Ministry of Health. National Population Health Survey 2019. (moh.gov.sg). Available online: https://www.moh.gov.sg/docs/librariesprovider5/default-document-library/nphs-2019-survey-report.pdf (accessed on 17 February 2021).
48. Kwon, D.S.; Kim, K.; Park, S.M. Factors associated with influenza vaccination coverage among the elderly in South Korea: The Fourth Korean National Health and Nutrition Examination Survey (KNHANES IV). *BMJ Open* **2016**, *6*, e012618. https://doi.org/10.1136/bmjopen-2016-012618.
49. González-Block, M. Ángel; Gutiérrez-Calderón, E.; Pelcastre-Villafuerte, B.E.; Arroyo-Laguna, J.; Comes, Y.; Crocco, P.; Fachel-Leal, A.; Noboa, L.; Riva-Knauth, D.; Rodríguez-Zea, B.; et al. Influenza vaccination hesitancy in five countries of South America. Confidence, complacency and convenience as determinants of immunization rates. *PLoS ONE* **2020**, *15*, e0243833. https://doi.org/10.1371/journal.pone.0243833.
50. Smith, G.J.; Bahl, J.; Vijaykrishna, D.; Zhang, J.; Poon, L.; Chen, H.; Webster, R.G.; Peiris, J.S.M.; Guan, Y. Dating the emergence of pandemic influenza viruses. *Proc. Natl. Acad. Sci. USA* **2009**, *106*, 11709–11712. https://doi.org/10.1073/pnas.0904991106.
51. Jones, R. How many extra deaths have really occurred in the UK due to the COVID-19 outbreak? Part LVIX. German states most affected by COVID-19. *Res. Gate* **2022**. https://doi.org/10.13140/RG.2.2.24965.22240.
52. Nielsen, J.; Krause, T.G.; Mølbak, K. Influenza-associated mortality determined from all-cause mortality, Denmark 2010/11–2016/17: The FluMOMO model. *Influenza Other Respir. Viruses* **2018**, *12*, 591–604. https://doi.org/10.1111/irv.12564.
53. Schanzer, D.L.; Sevenhuysen, C.; Winchester, B.; Mersereau, T. Estimating Influenza Deaths in Canada, 1992–2009. *PLoS ONE* **2013**, *8*, e80481. https://doi.org/10.1371/journal.pone.0080481.
54. CDC. CDC Seasonal Flu Vaccine Effectiveness Studies. CDC Seasonal Flu Vaccine Effectiveness Studies|CDC. Available online: https://www.cdc.gov/flu/vaccines-work/effectiveness-studies.htm (accessed on 25 January 2022).
55. CDC. Adult Obesity Facts. Adult Obesity Facts|Overweight & Obesity|CDC. Available online: https://www.cdc.gov/obesity/data/adult.html (accessed on 12 February 2022).
56. Achievements in Public Health, 1900–1999: Changes in the Public Health System. *JAMA* **2000**, *283*, 735–738. https://doi.org/10.1001/jama.283.6.735.
57. Woods, R. Mortality and sanitary conditions in the "Best governed city in the world"—Birmingham, 1870–1910. *J. Hist. Geogr.* **1978**, *4*, 35–56. https://doi.org/10.1016/0305–7488(78)90069–5.
58. Daniel, T.M. The history of tuberculosis. *Respir. Med.* **2006**, *100*, 1862–1870. https://doi.org/10.1016/j.rmed.2006.08.006.
59. Ledberg, A. A large decrease in the magnitude of seasonal fluctuations in mortality among elderly explains part of the increase in longevity in Sweden during 20th century. *BMC Public Health* **2020**, *20*, 1674. https://doi.org/10.1186/s12889-020-09749-4.
60. Gan, G.; Pang, J. Obesity in Singapore: Prevention and control. *Singap. Fam. Phys.* **2012**, *38*, 8–13.
61. Thomas, R.E. Are influenza-associated morbidity and mortality estimates for those ≥65 in statistical databases accurate, and an appropriate test of influenza vaccine effectiveness? *Vaccine* **2014**, *32*, 6884–6901. https://doi.org/10.1016/j.vaccine.2014.08.090.
62. Gagnon, A.; Miller, M.; Hallman, S.A.; Bourbeau, R.; Herring, D.A.; Earn, D.; Madrenas, J. Age-Specific Mortality During the 1918 Influenza Pandemic: Unravelling the Mystery of High Young Adult Mortality. *PLoS ONE* **2013**, *8*, e69586. https://doi.org/10.1371/journal.pone.0069586.
63. Grinbaum, E.; Ivanova, N.; Luzianina, T.; Nevedomskaia, G.; Kudriavtseva, V. Antigennaia i biologicheskaia kharakteristika virusov grippa A1, izolirovannykh v pandemiiu 1977 g [Antigenic and biological characteristics of influenza A1 viruses isolated in the 1977 pandemic]. *Vopr Virusol.* **1979**, *3*, 232–236.

64. Rozo, M.; Gronvall, G.K. The Reemergent 1977 H1N1 Strain and the Gain-of-Function Debate. *mBio* **2015**, *6*, e01013-15. https://doi.org/10.1128/mbio.01013-15.
65. Adalja, A.A.; Henderson, D. Original Antigenic Sin and Pandemic (H1N1) 2009. *Emerg. Infect. Dis.* **2010**, *16*, 1028.
66. Henry, C.; Palm, A.-K.E.; Krammer, F.; Wilson, P.C. From Original Antigenic Sin to the Universal Influenza Virus Vaccine. *Trends Immunol.* **2017**, *39*, 70–79. https://doi.org/10.1016/j.it.2017.08.003.
67. Wu, A.; Peng, Y.; Du, X.; Shu, Y.; Jiang, T. Correlation of Influenza Virus Excess Mortality with Antigenic Variation: Application to Rapid Estimation of Influenza Mortality Burden. *PLoS Comput. Biol.* **2010**, *6*, e1000882. https://doi.org/10.1371/journal.pcbi.1000882.
68. Gupta, V.; Earl, D.J.; Deem, M.W. Quantifying influenza vaccine efficacy and antigenic distance. *Vaccine* **2006**, *24*, 3881–3888. https://doi.org/10.1016/j.vaccine.2006.01.010.
69. Sitaras, I. Antigenic Cartography: Overview and Current Developments. *Methods Pharmacol. Toxicol.* **2020**, *2123*, 61–68. https://doi.org/10.1007/978-1-0716-0346-8_5.
70. Anderson, C.S.; McCall, P.R.; Stern, H.A.; Yang, H.; Topham, D.J. Antigenic cartography of H1N1 influenza viruses using sequence-based antigenic distance calculation. *BMC Bioinform.* **2018**, *19*, 51. https://doi.org/10.1186/s12859-018-2042-4.
71. Cai, Z.; Zhang, T.; Wan, X-F. Antigenic distance measurements for seasonal influenza vaccine selection. *Vaccine **2012***, *30*, 448–453. https://doi.org/10.1016/j.vaccine.2011.10.051.
72. Kawakami, C.; Yamayoshi, S.; Akimoto, M.; Nakamura, K.; Miura, H.; Fujisaki, S.; Pattinson, D.J.; Shimizu, K.; Ozawa, H.; Momoki, T.; et al. Genetic and antigenic characterisation of influenza A(H3N2) viruses isolated in Yokohama during the 2016/17 and 2017/18 influenza seasons. *Eurosurveillance* **2019**, *24*, 1800467. https://doi.org/10.2807/1560–7917.ES.2019.24.6.1800467.
73. CDC. Summary of the 2017–2018 Influenza Season. Summary of the 2017–2018 Influenza Season | CDC. Available online: https://www.cdc.gov/flu/about/season/flu-season-2017-2018.htm#:~:text=The%202017-2018%20influenza%20season%20was%20a%20high%20severity,geographically%20widespread%20influenza%20activity%20for%20an%20extended%20period. (accessed on 27 November 2021).
74. Thompson, W.W.; Moore, M.R.; Weintraub, E.; Cheng, P.-Y.; Jin, X.; Bridges, C.B.; Bresee, J.S.; Shay, D. Estimating Influenza-Associated Deaths in the United States. *Am. J. Public Health* **2009**, *99*, S225–S230. https://doi.org/10.2105/ajph.2008.151944.
75. CDC. Summary of the 2011–2012 Influenza Season. Summary of the 2011–2012 Influenza Season | CDC. Available online: https://www.cdc.gov/flu/pastseasons/1112season.htm (accessed on 15 December 2021).
76. CDC. Summary of the 2014–2015 Influenza Season. Summary of the 2014–2015 Influenza Season | CDC. Available online: https://www.cdc.gov/flu/pastseasons/1415season.htm (accessed on 15 December 2021).
77. CDC. Summary of the 2013–2014 Influenza Season. Summary of the 2013–2014 Influenza Season | CDC. Available online: https://www.cdc.gov/flu/pastseasons/1314season.htm#:~:text=During%20the%202013-2014%20season%2C%20CDC%20received%20reports%20of,were%20in%20people%2018%20to%2064%20years%20old. (accessed on 15 December 2021).
78. CDC. Disease Burden of Flu. Disease Burden of Flu | CDC. Available online: https://www.cdc.gov/flu/about/burden/index.html?web=1&wdLOR=c51D04AA5-C27A-4E2B-8344-9780EA08A310 (accessed on 15 December 2021).
79. CDC. Estimates of Deaths Associated with Seasonal Influenza—United States, 1976–2007. *Morb. Mortal. Wkly. Rep.* **2010**, *59*, 1057–1062.
80. CDC. How Many Adults Die from Flu Each Year? Frequently Asked Questions about Estimated Flu Burden | CDC. Available online: https://www.cdc.gov/flu/about/burden/faq.htm (accessed on 15 December 2021).
81. WHO. Introduction of Seasonal Influenza Vaccine. Introduction of Seasonal Influenza Vaccine (who.int). Available online: https://immunizationdata.who.int/pages/vaccine-intro-by-antigen/influenza.html (accessed on 16 January 2022).
82. Walzer, P.; Estève, C.; Barben, J.; Menu, D.; Cuenot, C.; Manckoundia, P.; Putot, A. Impact of Influenza Vaccination on Mortality in the Oldest Old: A Propensity Score-Matched Cohort Study. *Vaccines* **2020**, *8*, 356. https://doi.org/10.3390/vaccines8030356.
83. Rizzo, C.; Viboud, C.; Montomoli, E.; Simonsen, L.; Miller, M.A. Influenza-related mortality in the Italian elderly: No decline associated with increasing vaccination coverage. *Vaccine* **2006**, *24*, 6468–6475. https://doi.org/10.1016/j.vaccine.2006.06.052.
84. Head, A.; Fleming, K.; Kypridemos, C.; Schofield, P.; Pearson-Stuttard, J.; O'Flaherty, M. Inequalities in incident and prevalent multimorbidity in England, 2004–2019: A population-based, descriptive study. *Lancet Health Longev.* **2021**, *2*, e489–e497. https://doi.org/10.1016/s2666-7568(21)00146-x.
85. Lebenbaum, M.; Zaric, G.S.; Thind, A.; Sarma, S. Trends in obesity and multimorbidity in Canada. *Prev. Med.* **2018**, *116*, 173–179. https://doi.org/10.1016/j.ypmed.2018.08.025.

86. McElhaney, J.E.; Kuchel, G.; Zhou, X.; Swain, S.L.; Ehaynes, L. T-Cell Immunity to Influenza in Older Adults: A Pathophysiological Framework for Development of More Effective Vaccines. *Front. Immunol.* **2016**, *7*, 41. https://doi.org/10.3389/fimmu.2016.00041.
87. Gutiérrez-González, E.; Cantero-Escribano, J.M.; Redondo-Bravo, L.; Juan-Sanz, I.S.; Robustillo-Rodela, A.; Cendejas-Bueno, E. Influenza Working Group Effect of vaccination, comorbidities and age on mortality and severe disease associated with influenza during the season 2016–2017 in a Spanish tertiary hospital. *J. Infect. Public Health* **2019**, *12*, 486–491. https://doi.org/10.1016/j.jiph.2018.11.011.
88. Preval, N.; Keall, M.; Telfar-Barnard, L.; Grimes, A.; Howden-Chapman, P. Impact of improved insulation and heating on mortality risk of older cohort members with prior cardiovascular or respiratory hospitalisations. *BMJ Open* **2017**, *7*, e018079. https://doi.org/10.1136/bmjopen-2017-018079.
89. WHO. Housing and Health Guidelines. Chapter 4: Low Indoor Temperatures and Insulation. 2018. Low Indoor Temperatures and Insulation—WHO Housing and Health Guidelines—NCBI Bookshelf (nih.gov). Available online: https://www.ncbi.nlm.nih.gov/books/NBK535294/ (accessed on 15 December 2021).
90. Huebner, G.M.; Hamilton, I.; Chalabi, Z.; Shipworth, D.; Oreszczyn, T. Comparison of indoor temperatures of homes with recommended temperatures and effects of disability and age: An observational, cross-sectional study. *BMJ Open* **2018**, *8*, e021085. https://doi.org/10.1136/bmjopen-2017-021085.
91. Saeki, K.; Obayashi, K.; Iwamoto, J.; Tone, N.; Okamoto, N.; Tomioka, K.; Kurumatani, N. Stronger association of indoor temperature than outdoor temperature with blood pressure in colder months. *J. Hypertens.* **2014**, *32*, 1582–1589. https://doi.org/10.1097/hjh.0000000000000232.
92. NICE. Excess Winter Deaths and Illness and the Health Risks Associated with Cold Homes. NICE Guideline [NG6], 5 March 2015. Overview|Excess Winter Deaths and Illness and the Health Risks Associated with Cold Homes|Guidance|NICE. Available online: https://www.nice.org.uk/guidance/NG6 (accessed on 29 December 2021).
93. Hajat, S. Evidence Review and Economic Analysis of Excess Winter Deaths. 2015. Available online: https://www.nice.org.uk/guidance/ng6/evidence/evidence-review-2-interventions-and-economic-studies-pdf-544621934 (accessed on 29 December 2021).
94. Fyfe, C.; Telfar, L.; Barnard; Howden-Chapman, P.; Douwes, J. Association between home insulation and hospital admission rates: Retrospective cohort study using linked data from a national intervention programme. *BMJ* **2020**, *371*, m4571. https://doi.org/10.1136/bmj.m4571.
95. Oktora, M.P.; Denig, P.; Bos, J.H.J.; Schuiling-Veninga, C.C.M.; Hak, E. Trends in polypharmacy and dispensed drugs among adults in the Netherlands as compared to the United States. *PLoS ONE* **2019**, *14*, e0214240. https://doi.org/10.1371/journal.pone.0214240.
96. Agarwal, D.; Schmader, K.E.; Kossenkov, A.V.; Doyle, S.; Kurupati, R.; Ertl, H.C.J. Immune response to influenza vaccination in the elderly is altered by chronic medication use. *Immun. Ageing* **2018**, *15*, 19. https://doi.org/10.1186/s12979-018-0124-9.
97. Iloanusi, S.; Mgbere, O.; Essien, E.J. Polypharmacy among COVID-19 patients: A systematic review. *J. Am. Pharm. Assoc.* **2021**, *61*, e14–e25. https://doi.org/10.1016/j.japh.2021.05.006.
98. Frazier, S.C. Health Outcomes and Polypharmacy in Elderly Individuals. *J. Gerontol. Nurs.* **2005**, *31*, 4–9. https://doi.org/10.3928/0098-9134-20050901-04.
99. Lin, C.-J.; Chang, Y.-C.; Tsou, M.-T.; Chan, H.-L.; Chen, Y.-J.; Hwang, L.-C. Factors associated with hospitalization for community-acquired pneumonia in home health care patients in Taiwan. *Aging Clin. Exp. Res.* **2019**, *32*, 149–155. https://doi.org/10.1007/s40520-019-01169-8.
100. Sheridan, P.A.; Paich, H.A.; Handy, J.; Karlsson, E.A.; Hudgens, M.G.; Sammon, A.B.; Holland, L.A.; Weir, S.; Noah, T.L.; Beck, M.A. Obesity is associated with impaired immune response to influenza vaccination in humans. *Int. J. Obes.* **2012**, *36*, 1072–1077. https://doi.org/10.1038/ijo.2011.208.
101. Frasca, D.; Ferracci, F.; Diaz, A.; Romero, M.; Lechner, S.; Blomberg, B.B. Obesity decreases B cell responses in young and elderly individuals. *Obesity* **2016**, *24*, 615–625. https://doi.org/10.1002/oby.21383.
102. Frasca, D.; Diaz, A.; Romero, M.; Blomberg, B.B. Phenotypic and Functional Characterization of Double Negative B Cells in the Blood of Individuals With Obesity. *Front. Immunol.* **2021**, *12*, 98. https://doi.org/10.3389/fimmu.2021.616650.
103. Frasca, D.; Reidy, L.; Romero, M.; Diaz, A.; Cray, C.; Kahl, K.; Blomberg, B.B. The majority of SARS-CoV-2-specific antibodies in COVID-19 patients with obesity are autoimmune and not neutralizing. *Int. J. Obes.* **2021**, *46*, 427–432. https://doi.org/10.1038/s41366-021-01016-9.
104. Livio, L.; Radaelli, M. Influenza and obesity: Its odd relationship and the lessons for COVID-19 pandemic. *Acta Diabetol.* **2020**, *57*, 759–764.
105. CDC. Current Cigarette Smoking among Adults in the United States. Current Cigarette Smoking Among Adults in the United States|CDC. Available online: https://www.cdc.gov/tobacco/data_statistics/fact_sheets/adult_data/cig_smoking/index.htm (accessed on 24 December 2021).
106. Chavez, J.; Hai, R. Effects of Cigarette Smoking on Influenza Virus/Host Interplay. *Pathogens* **2021**, *10*, 1636. https://doi.org/10.3390/pathogens10121636.

107. Godoy, P.; Castilla, J.; Soldevila, N.; Mayoral, J.M.; Toledo, D.; Martín, V.; Astray, J.; Egurrola, M.; Morales-Suarez-Varela, M.; Domínguez, A. Smoking may increase the risk of influenza hospitalization and reduce influenza vaccine effectiveness in the elderly. *Eur. J. Public Health* **2017**, *28*, 150–155. https://doi.org/10.1093/eurpub/ckx130.
108. Corrada, M.M.; Brookmeyer, R.; Paganini-Hill, A.; Berlau, D.; Kawas, C.H. Dementia incidence continues to increase with age in the oldest old: The 90+ study. *Ann. Neurol.* **2010**, *67*, 114–121. https://doi.org/10.1002/ana.21915.
109. Brandeis, G.H.; Berlowitz, D.; Coughlin, N. Mortality Associated with an Influenza Outbreak on a Dementia Care Unit. *Alzheimer Dis. Assoc. Disord.* **1998**, *12*, 140–145. https://doi.org/10.1097/00002093-199809000-00004.
110. Pawelec, G.; McElhaney, J. Recent advances in influenza vaccines. *F1000Research* **2020**, *9*, 305. https://doi.org/10.12688/f1000research.22611.1.
111. Tsilibary, E.-P.; Charonis, S.A.; Georgopoulos, A.P. Vaccines for Influenza. *Vaccines* **2021**, *9*, 47. https://doi.org/10.3390/vaccines9010047.
112. Cheng, Y.J.; Imperatore, G.; Geiss, L.S.; Wang, J.; Saydah, S.H.; Cowie, C.C.; Gregg, E.W. Secular Changes in the Age-Specific Prevalence of Diabetes Among U.S. Adults: 1988–2010. *Diabetes Care* **2013**, *36*, 2690–2696. https://doi.org/10.2337/dc12-2074.
113. Valdez, R.; Narayan, K.M.; Geiss, L.S.; Engelgau, M.M. Impact of diabetes mellitus on mortality associated with pneumonia and influenza among non-Hispanic black and white US adults. *Am. J. Public Health* **1999**, *89*, 1715–1721. https://doi.org/10.2105/ajph.89.11.1715.
114. Wang, C.-S.; Wang, S.-T.; Lai, C.-T.; Lin, L.-J.; Chou, P. Impact of influenza vaccination on major cause-specific mortality. *Vaccine* **2007**, *25*, 1196–1203. https://doi.org/10.1016/j.vaccine.2006.10.015.
115. Schade, C.P.; McCombs, M.A. Influenza immunization and mortality among diabetic Medicare beneficiaries in West Virginia. *West Va Med. J.* **2003**, *96*, 444–448.
116. White, M.C.; Holman, D.M.; Boehm, J.E.; Peipins, L.A.; Grossman, M.; Henley, S.J. Age and Cancer Risk: A Potentially Modifiable Relationship. *Am. J. Prev. Med.* **2014**, *46*, S7–S15. https://doi.org/10.1016/j.amepre.2013.10.029.
117. Carreira, H.; Strongman, H.; Peppa, M.; McDonald, H.I.; Dos-Santos-Silva, I.; Stanway, S.; Smeeth, L.; Bhaskaran, K. Prevalence of COVID-19-related risk factors and risk of severe influenza outcomes in cancer survivors: A matched cohort study using linked English electronic health records data. *eClinicalMedicine* **2020**, *29–30*, 100656. https://doi.org/10.1016/j.eclinm.2020.100656.
118. Shehata, M.A.; Karim, N.A. Influenza Vaccination in Cancer Patients Undergoing Systemic Therapy. *Clin. Med. Insights: Oncol.* **2014**, *8*, 57–64. https://doi.org/10.4137/cmo.s13774.
119. Chen, H.; Kaufman, J.S.; Olaniyan, T.; Pinault, L.; Tjepkema, M.; Chen, L.; van Donkelaar, A.; Martin, R.V.; Hystad, P.; Chen, C.; et al. Changes in exposure to ambient fine particulate matter after relocating and long term survival in Canada: Quasi-experimental study. *BMJ* **2021**, *375*, 2368. https://doi.org/10.1136/bmj.n2368.
120. Glencross, D.A.; Ho, T.-R.; Camiña, N.; Hawrylowicz, C.M.; Pfeffer, P.E. Air pollution and its effects on the immune system. *Free Radic. Biol. Med.* **2020**, *151*, 56–68. https://doi.org/10.1016/j.freeradbiomed.2020.01.179.
121. Lindner-Cendrowska, K.; Bröde, P. Impact of biometeorological conditions and air pollution on influenza-like illnesses incidence in Warsaw. *Int. J. Biometeorol.* **2021**, *65*, 929–944. https://doi.org/10.1007/s00484-021-02076-2.
122. Lu, B.; Wang, Y.; Zhu, Z.; Zhang, Z.; Dong, T.; Li, F.; Gao, Y.; Du, X.; Qu, Z. Epidemiological and genetic characteristics of influenza virus and the effects of air pollution on laboratory-confirmed influenza cases in Hulunbuir, China, from 2010 to 2019. *Epidemiol. Infect.* **2020**, *148*, e159. https://doi.org/10.1017/s0950268820001387.
123. Wong, C.M.; Yang, L.; Thach, T.Q.; Chau, P.Y.K.; Chan, K.P.; Thomas, G.N.; Lam, T.H.; Wong, T.W.; Hedley, A.J.; Peiris, J.M. Modification by Influenza on Health Effects of Air Pollution in Hong Kong. *Environ. Health Perspect.* **2009**, *117*, 248–253. https://doi.org/10.1289/ehp.11605.
124. Liu, K.; Yang, B.-Y.; Guo, Y.; Bloom, M.S.; Dharmage, S.; Knibbs, L.D.; Heinrich, J.; Leskinen, A.; Lin, S.; Morawska, L.; et al. The role of influenza vaccination in mitigating the adverse impact of ambient air pollution on lung function in children: New insights from the Seven Northeastern Cities Study in China. *Environ. Res.* **2020**, *187*, 109624. https://doi.org/10.1016/j.envres.2020.109624.
125. ONS. Deaths by Single Year of Age Tables, UK—2020—GOV.UK (www.gov.uk). Available online: https://www.gov.uk/government/statistics/deaths-by-single-year-of-age-tables-uk-2020 (accessed on 21 February 2022).
126. Yin, Q.; Wang, J.; Ren, Z.; Li, J.; Guo, Y. Mapping the increased minimum mortality temperatures in the context of global climate change. *Nat. Commun.* **2019**, *10*, 4640. https://doi.org/10.1038/s41467-019-12663-y.
127. Allen, M.J.; Sheridan, S.C. Mortality risks during extreme temperature events (ETEs) using a distributed lag non-linear model. *Int. J. Biometeorol.* **2015**, *62*, 57–67. https://doi.org/10.1007/s00484-015-1117-4.
128. Office for National Statistics. Excess Winter Mortality in England and Wales: 2012 to 2013 (Provisional) and 2011 to 2012 (Final). 26 November 2013. (publishing.service.gov.uk) Available online:

https://assets.publishing.service.gov.uk/government/uploads/system/uploads/attachment_data/file/229819/Excess_winter_mortality_2012.pdf#:~:text=Excess%20winter%20mortality%202012-13%20Excess%20all-cause%20mortality%20has,Diseases%20Department%20%28RDD%29%20within%20Public%20Health%20England%20%28PHE%29 (accessed on 15 December 2021).
129. Murtas, R.; Russo, A.G. Effects of pollution, low temperature and influenza syndrome on the excess mortality risk in winter 2016–2017. *BMC Public Health* **2019**, *19*, 1445. https://doi.org/10.1186/s12889-019-7788-8.
130. Rosano, A.; Bella, A.; Gesualdo, F.; Acampora, A.; Pezzotti, P.; Marchetti, S.; Ricciardi, W.; Rizzo, C. Investigating the impact of influenza on excess mortality in all ages in Italy during recent seasons (2013/14–2016/17 seasons). *Int. J. Infect. Dis.* **2019**, *88*, 127–134. https://doi.org/10.1016/j.ijid.2019.08.003.
131. Thurner, S.; Klimek, P.; Hanel, R. *Introduction to the Theory of Complex Systems*; Oxford University Press (OUP): Oxford, UK, 2018.
